# Recent Progress on Microelectrodes in Neural Interfaces

**DOI:** 10.3390/ma11101995

**Published:** 2018-10-16

**Authors:** Geon Hwee Kim, Kanghyun Kim, Eunji Lee, Taechang An, WooSeok Choi, Geunbae Lim, Jung Hwal Shin

**Affiliations:** 1 Department of Mechanical Engineering, Pohang University of Science and Technology, Pohang 37673, Korea; q16164545@postech.ac.kr (G.H.K.); qwnerkang@postech.ac.kr (K.K.); eunji4993@postech.ac.kr (E.L.); 2 Department of Mechanical Design Engineering, Andong National University, Kyungbuk 760-749, Korea; tcmerias@anu.ac.kr; 3 Department of Mechanical Engineering, Korea National University of Transportation, Chungju 380-702, Korea; w.choi@ut.ac.kr; 4 School of Mechanical Engineering, Kyungnam University, Changwon 51767, Korea

**Keywords:** neural electrode, brain‒machine interface (BMI), microelectrode array (MEA), chronically implanted neural electrodes, electroencephalogram (EEG) electrode, surface modification

## Abstract

Brain‒machine interface (BMI) is a promising technology that looks set to contribute to the development of artificial limbs and new input devices by integrating various recent technological advances, including neural electrodes, wireless communication, signal analysis, and robot control. Neural electrodes are a key technological component of BMI, as they can record the rapid and numerous signals emitted by neurons. To receive stable, consistent, and accurate signals, electrodes are designed in accordance with various templates using diverse materials. With the development of microelectromechanical systems (MEMS) technology, electrodes have become more integrated, and their performance has gradually evolved through surface modification and advances in biotechnology. In this paper, we review the development of the extracellular/intracellular type of in vitro microelectrode array (MEA) to investigate neural interface technology and the penetrating/surface (non-penetrating) type of in vivo electrodes. We briefly examine the history and study the recently developed shapes and various uses of the electrode. Also, electrode materials and surface modification techniques are reviewed to measure high-quality neural signals that can be used in BMI.

## 1. Introduction

Neural electrode is an interface between neurons—the electroactive cells of the nervous system—and brain-machine interface (BMI) system. They are used in basic neuroscience to facilitate our comprehension of physiological processes at the cellular level, and in BMI to substitute for function of the injured nervous system. The purpose of neural electrodes is to record neural signals with high signal-to-noise ratios (SNRs) from several individual neurons, termed action potentials (APs) [1]. To realize BMI, several processes are enacted, such as the recording of neural signals, analysis of recorded neural signals, implementation of algorithms for robot control, and robot control using neural signals. The recording of neural signals by neural electrodes provides a starting point for various applications and, as such, neural electrodes are key to the success of these endeavors.

In vitro studies contribute to our understanding of the electric connections between the neurons and the function of the neurons in the nervous system. The cultured neurons emit the electrical signals, which are construed as the neural and physiological information. To record the electrical signal, the in vitro neural electrodes array, so called microelectrode arrays (MEAs), are commonly used. Also, to confirm the validity of neural electrode designs and recording techniques, the electric signals from animals, such as guinea pigs, rats or monkeys, are recorded in vivo.

The in vivo electrodes are divided according to their position at the time of the signal’s recording. Selection of the appropriate neural electrode is crucial, considering the trade-off between the quality of the recorded signal and the degree of invasion. Generally, neural electrodes that are implanted into the brain have higher SNRs than EEG electrodes, and are classified according to the method used to fabricate the probe that detects the AP. Currently, polymer-based electrodes with low Young’s modulus are favored. The EEG electrodes are aimed at capturing the EEG signal, and may be classified as wet, dry, or non-contact, with further sub-categorization that pertains to the level of invasion with which they are associated. While the material used for fabricating the electrodes is a key aspect of their functionality, recent studies have focused on their flexibility and elasticity, in the interest of addressing the problem of the electrodes’ positioning.

Electrodes for measuring neural signals necessarily make direct contact with the body, whether they are of the penetrating or non-penetrating type. Thus, all substances used in fabricating neural electrodes should be of types that do not trigger immune response. Additionally, the nerve electrode must maintain contact with the body over a long period of time, and the substance must be denatured so as not to emit toxins [2].

Several investigations have been conducted into the interface between the electrode and the anatomical part, in addition to the material from which the electrode has been fabricated. In this interface, intracellular/extracellular signal is transferred via electrochemical reaction, and the electrode itself is broken down by long-term measurement. For these reasons, numerous studies have sought to improve electrode performance by applying metal coating or nanostructure synthesis to electrode surfaces, in what is known as the surface modification technique.

Generally speaking, selectivity and sensitivity are the two most important factors in evaluating biosensor performance [3]. With regard to neurons, selectivity means spatial resolution, while sensitivity refers to slight changes in the potential of the target cell/brain tissue. Therefore, both selectivity and sensitivity must be maximized for optimum signal receptivity. For example, as the exposed area of the nerve electrode increases, the electrical impedance is decreased, but the spatial resolution deteriorates simultaneously. As the exposed area decreases, the spatial resolution improves, but this is associated with an increase in electrical impedance [4]. Consequently, several studies have sought to maximize the electrochemical surface area (ESA) of electrodes, while maintaining a smaller geometrical surface area (GSA) [5].

Neural electrodes are widely used not only for measuring neural signals but also for stimulating neurons. Electrical brain stimulation was first used in the first half of the 19th century to stimulate a neuron or neural network in the brain with an electric current. Although this method has been widely used for a long time, it has several critical drawbacks. The electrical stimulation uses an electric current between two electrodes, the focal stimulation on the neuronal population is difficult, and it can cause brain damage due to the current. Thus, there has been interest in overcoming these drawbacks and developing alternative techniques to trigger neuronal activity [6,7].

This review focuses on: (1) electrodes for measuring neural signals in an in vitro environment; (2) penetrating electrodes for measuring neural signals in an in vivo environment; (3) non-penetrating electrodes for measuring neural signals in an in vivo environment; (4) surface modification techniques for improved neural signal recording.

In this paper, we review the role of electrodes in recording electrical nerve signals transmitted between nerves, and the problems that may arise in the transmission of electrical nerve signals between nerves. In addition, we examine the various types and characteristics of electrodes developed to solve them. By reviewing these strategies, we expect to be able to understand the flow of electrode development.

## 2. Microelectrode Array (MEA) for In Vitro Applications

Neural interface is an important tool for enhancing our understanding of the nervous system, owing to its capacity for stimulating neural cells and recording neural signals [8]. Among the various types of neural interface, MEA is of the utmost significance, as it is used to measure the activity signals emitted by cultured neuronal cells in several neuroscientific applications. Full appreciation of the mechanisms that underlie the electrical connections between neurons within their networks is crucial in neuroscience, for exploring the functionality of neurons within the nervous system. Cellular recording using MEA has been employed for measuring AP, and for processing neural information in neural circuits.

Two types of measurement use MEA: extracellular and intracellular recordings. Extracellular recording is a less invasive method capable of performing long-term recording; extracellular signals are field potentials and poor in quality, however, compared with intracellular signals. While intracellular recording utilizes synaptic potentials and more sensitive, it is associated with cell damage more, as it is a more invasive method [8,9]. Accordingly, methods are carefully selected depending on the purpose behind signal recording, and recording methods have been developed. For precise AP recording, the MEA should adhere well to the target cells, and the contact surface for a single unit cell should not exceed 4000 µm^2^ [5]. Furthermore, the impedance of MEA should be small, as the SNR has an inverse relationship with electrode impedance [5].

It is generally planar MEA that is used in in vitro experiments, and it has been progressively developed and commercialized. Recently, other MEA types have received increasing attention. Extracellular electrodes utilize microstructures adopting a biomimetic approach that incorporates the shape of the dendritic spine, so that the electrode may be surrounded by the cell membrane [10]. Intracellular electrodes utilize nanostructures adopting a biocompatibility approach, which may have lower resistance properties, similar to nanotube microelectrodes and nanowire microelectrodes [4]. Recently, researchers have developed a flexible and stretchable electrode by changing the substrate material instead of the shape of electrode surface, for applying to in vivo experiment on neural interfaces [11].

### 2.1. History

The use of neural interfaces as recording devices commenced toward the end of the 1930s, when the size of neural interfaces diminished, and developments led to the possibility of recording both interior and exterior electrical potential signals, via a single neural fiber immersed in conducting fluid [12]. To record action potential signals simultaneously from different neuronal units, a high-throughput recording interface with the nervous system is required. In line with improvements in manufacturing methods, structure and materials, Robinson introduced the first metal microelectrode in 1968 [13], and in 1972, Thomas et al. introduced the first MEA [14]. This device could record local field potential (LFP) signals from spontaneous contracting array that consisted of platinized gold microelectrodes embedded onto a glass substrate. However, the device was unable to record activity signals from a single cell. Following improvements to the electrode configuration, the APs of excitable cells could be measured [15]. Some researchers have reported that biocompatible materials, such as carbon nanotubes (CNTs), may provide a suitable surface for neuronal cell adhesion and growth, regardless of surface uniformity [16,17]. CNTs are suitable for fabricating electrodes as they have biocompatibility and share a similar minute nature with bio-membrane. Recently, Hai et al. recommended gold spine-shaped electrodes that are associated with enhanced signal-recording quality [10]. Similarly, the shape of MEA has developed such that it can firmly grasp the neuronal membrane, adopting a biomimetic approach, to attain subthreshold synaptic potential similar to the APs [10].

### 2.2. Type of MEA

#### 2.2.1. MEA for Extracellular Recording

##### Planar Type MEA

**Figure 1:**
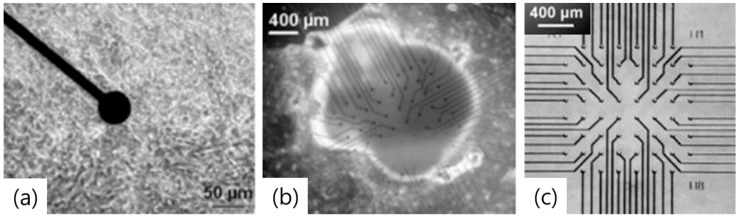
(**a**) Myocytes of embryonic chicken ventricles are cultured on the planar type MEA as cell culture dish [20]. (**b**) A whole embryonic heart is kept on a MEA [20]. (**c**) Each electrode is connected with a contact pad by conductor lines [20]. Copyright 2003, Springer-Verlag.

Planar-type MEA is widely used as a cell culture dish for investigating network activity (Figure 1a,b). Its electrodes are composed of nontoxic and anti-corrosive metals, such as gold, titanium nitride, and platinum. Although glass and silicon wafers are both viable options for constructing the substrate, glass wafers are more usually selected, as they permit easy observation of cultured cells using conventional transmitted light microscopy. To improve cell adhesion, the substrate is commonly coated with poly-l-lysine or laminin. The electrodes are insulated from one another, using inorganic or organic materials, such as epoxy resin or silicon oxide, are they are embedded within the culture medium. A single electrode has recording site of the range of a few tens of micrometers equivalent in size to a single cell with sufficiently low impedance. When positioned in high-density arrays, it provides the best signal-to- noise ratio and high spatial resolution. Each electrode is connected to a contact pad via thin contactors (conductor lines) for recording extracellular signals (Figure 1c) [18]. Individual contact pads should be smaller than the cells to record the activity of a single cell, but reduction of the contact area increases the electrode impedance, resulting in increased signal noise [19]. Currently, the standard size of a gold electrode is reportedly 60 µm in diameter. During the last decade, planar-type MEA has undergone significant commercialization.

##### Biomimetic MEA

Spira et al. reported an extracellular neural interface adopting a biomimetic approach in the late 2000s [10]. Previously existing extracellular recording electrodes could achieve non-invasive and long-term recording; however, the signal obtained was generally weak and of inferior quality [20] The developed microelectrodes are equipped to overcome the disadvantages of extracellular recording as long as 2 days, while retaining its advantages. They are non-invasive and capable of long-term recording, consistently receiving strong, high-quality signals. In 2007, Spira et al. designed electrodes with micrometer-sized nail structures that protrude from the sensing pad, imitating dendritic spines [21]. Improvements in recording signals are mainly attributable to the electrodes’ unique shape, which facilitates their being engulfed by neurons, ensuring that they play a prominent role in signal transmission at excitatory synapses [22]. This enhances the junctional membrane conductance, and the seal resistance between the electrode and cell membrane [8]. Furthermore, the electrical coupling coefficient between neurons and the gold dendritic spine-shaped electrodes is increased to 50%, compared with that of the gold planar MEA, which is 0.1%. Consequently, the dendritic spine shaped electrode can record subthreshold synaptic potentials, in addition to the APs, similar to the recording properties of intracellular glass electrodes.

#### 2.2.2. MEA for Intracellular Recording

##### Sharp Glass Electrodes/Patch-Clamp Electrodes

**Figure 2:**
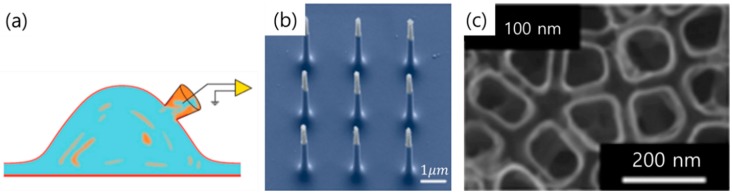
(**a**) Whole cell patch electrode configuration [8]. Copyright 2013, Nature Publication; (**b**) SEM image of the vertical nanowire electrode arrays [29]. Copyright 2012, Nature Publication; (**c**) TiO_2 nanotube arrays with 100 nm diameter [26]. Copyright 2007, ACS Publication.

Reduction of the junctional membrane resistance is crucial. This is, in fact, the approach conventionally adopted in classical methods using sharp electrodes [23]. Sharp glass electrodes and patch-clamp electrodes (Figure 2a) offer excellent intracellular recording by penetrating the plasma membrane and directly accessing the cytosol, while generating effective seal impedance with the plasma membrane (hundreds of MΩ to a few GΩ, respectively) [24]. However, among this method’s disadvantages are its reliance on bulky micromanipulators to steer the electrode tips, and its limitation on multi recording signals from a single cell at a time. Furthermore, intracellular recording has a very short duration, owing to biophysical instability.

Recently, Martina has demonstrated this approach’s potential for adaptation to neuronal networks [25]. Neurons were co-cultured on the electrode, and subsequently cell bodies formed chemical synapses and adhered to the substrate, achieving very high seal resistance (GΩ). This patch configuration affords neurons greater stability than do classical configurations.

##### Vertical Electrodes Using Nanostructure (Nanowire/Nanotube)

Vertical electrodes are capable of achieving intracellular recording with high SNR, due to their high aspect ratio [26]. During recording of the brain’s neural circuit activities, accurate monitoring and minute controlling are important [27]. Accordingly, the efficiency of signal recording is affected by electrical coupling and the contact between the cell and the electrode surface. Therefore, the electrodes should be high in density and small in size [28]. Vertical electrodes satisfy both of these conditions and are, thus, attracting attention.

One example of the vertical nanowire electrode arrays(VNEAs) were constructed from a doped silicon core encapsulated by silicon dioxide and tipped by Ti/Au (Figure 2b) [29]. Neurons were cultured on the VNEA for several days, and subsequently around 50% of the VNEAs penetrated through the plasma membrane of the neurons. Consequently, the recording properties of VNEAs have the advantage that the maximum record amplitude achieved from an individual neuron is 100% higher than that of the planar-type electrode. Figure 2c illustrates a nanotube electrode array. Neurons were cultured on the nanotube electrode array and, subsequently, the cell membrane was wrapped around and extended into the pore center of the array. The nanotube electrode arrays with diameter of 100 μm featured electrochemical impedance of 14 kΩ, which is about 10,000 times smaller than the VNEAs, and charge storage 4.91 mC/cm^2^ higher than that of the VNEAs [30]. Moreover, it could record large signals with high stability [31].

### 2.3. Challenges

Over the past several decades, studies have indicated that MEA performance would be significantly enhanced through structure control, extending to shape, size and materials. Nanostructured electrodes have exhibited the potential for stable signal recording, due to their large surface area and small size. As existing neural interfaces still have limitations, including high impedance, large size and elevated cell-damage threat, attempts to develop enhanced neural interfaces are ongoing.

Cultured neuronal networks have been used as in vitro model systems in many fields of neuroscience, including axon guidance [32], neural plasticity [33], nerve regeneration [34], and synaptogenesis [35]. To keep pace with future developments in neuroscience related to signal recording, improvements in the field of MEA may be facilitated through addressing the following aspects: (1) integrated porous coatings to increase biocompatibility and facilitate drug delivery; (2) combined recording of intracellular and extracellular electrical activity to promote research in computational neuroscience; (3) an electrode interface capable of maintaining constant impedance; (4) a multiplexed array of numerous electrodes that can record high SNR electrical activity from neural tissues, with high spatial and temporal resolution over long periods of time; (5) upscaling of multiplexed electrode arrays to higher channel counts, with a relatively small increase in the total number of readout wires.

## 3. Penetrating Electrode for In Vivo Applications

Chronically implanted neural electrodes are surgically implanted into the cortex or white matter, to record the APs of their neurons. Although these electrodes comprise a recording component and another component for integration and signal delivery, the recording component’s design is critical and usually includes small needles designed to penetrate the brain. For recording, a conductive microwire with only its tip exposed, and a sharp electrode made of MEMS technology, are used individually, or several are integrated.

**Figure 3:**
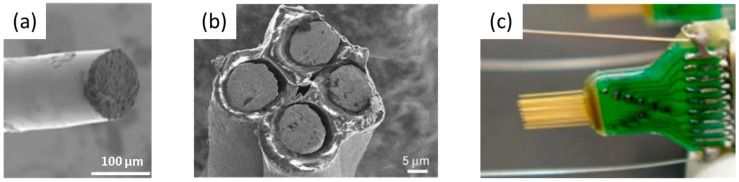
The types of microwire neural electrode (**a**) single wire [38], Copyright 2017, Elsevier; (**b**) Tetrode [39], Copyright 2017, Elsevier; (**c**) multi-wire electrode [40], Copyright 2010, Frontiers.

Generally speaking, neural signals recorded using a chronically-implanted neural electrode yield more information than non-invasive type electrodes [36]. To obtain an AP recording of superior quality, the electrode should be placed adjacent to neurological cells, with the area of the single exposed conductive part kept below 4000 μm^2^ with an SNR of 5 or more [37]. As the electrodes are intended for long-term implantation, it is imperative that toxicity, and mechanical and electrical properties are borne in mind when the electrode and insulating materials are selected. Herein, the chronically-implanted neural electrodes are classified according to each electrode’s shape and its material of fabrication (Figure 3).

### 3.1. History

BCI is aimed at elucidating the mechanisms by which information is transmitted between neurons, and investigating associated medical procedures and devices, such as deep brain stimulation and cochlear implants. BCI research began in 1929 when the EEG signals in the human brain were recorded for the first time [41]. Subsequent to this, attempts to record electrical signals in the human thalamus were made in 1949 [42], and a neural probe fabricated from tungsten was applied to a cat in 1959 [43]. In 1961, as research began to flourish in earnest, a multi-wire electrode was introduced to a human for the first time [44]. In 1963, a tungsten wire was used to measure the AP of a single cell [45]. By 1970, silicon-based electrodes had been developed [46] and, as a result of improvements in integration methods, in 1981, they were expanded to thirty channels [47]. Experimental progress also advanced to investigate various aspects of memory [48], arm control [49], and language [50]. In 1991, the so-called “Utah” electrode was developed and incorporated into experiments [51], and since 2001, polymer-based flexible electrodes have emerged. Since that time, studies in substrate material and post-fabrication functionalization have been ongoing.

### 3.2. Shape of Each Electrode

#### 3.2.1. Microwire Type

Microwire-type electrodes constitute small metal wires with insulated tips of less than a few hundred micrometers in diameter. Microwire-types can be sub-classified into single-wire [52], tetrode [53], and multi-wire [54] electrodes, depending on the number of electrodes used and the recording target; single-wire is used for intracellular recording, while tetrode and multi-wire are used for extracellular recording. Non-toxic metals, with good corrosion resistance, are used to fabricate the electrodes, including platinum [55], iridium [56], stainless steel [57], and alloys of these [58,59]. Recently, carbon-based materials have been used. Shin et al. fabricated the carbon nanotube electrode with an impedance per-unit area of 99.92% less than that made from tungsten [52]. The strengths of this design are that it is easily fabricated [60], and that it permits a high degree of freedom in assembly, and so it is cost-efficient and convenient. 

As long as the microwire has been in use, various animals, including rats [61] and guinea pigs [62] have been implanted and recorded using it. Kruger et al. went so far as to record the AP of a monkey [63] over 7 years at one-third of electrodes. However, serious problems remain in the shape disparities between the fabricated electrodes and isolation regions [61], the compromising of positional accuracy due to buckling during implantation [64], and compression of the underlying tissue [65]. Additionally, failure of the insulation material, peeling and corrosion of metal [61] and signal changes due to inflammation [66] also need to be addressed.

#### 3.2.2. Microelectrodes

**Figure 4:**
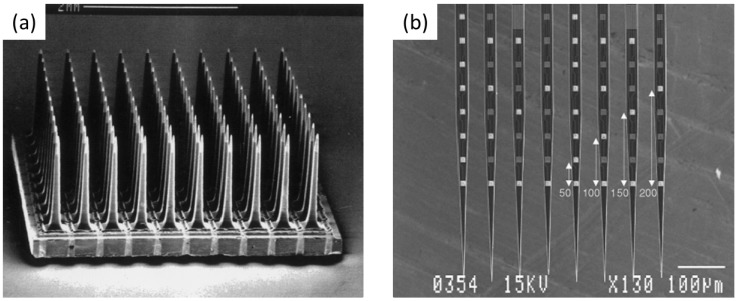
Representative microelectrode (**a**) Utah electrode [70], Copyright 1999, Elsevier; (**b**) Michigan electrode [71], Copyright 2004, Elsevier.

Advances in MEMS and micromachining technology have resulted in the development of silicon-based neural electrodes [67]. These technologies achieved miniaturization and uniformity of electrode size, which cannot be realized in microwire electrodes. Consequently, the electrodes could be integrated with associated improvements in signal reliability. Furthermore, owing to the production of rigid materials at wafer-scale, the problem of buckling was reduced and mass production was facilitated, so that APs could be recorded in several spaces, and the brain could be accurately mapped. Various neural electrode designs have been developed through the application of various materials and adjustments to the process, and these electrodes have successfully measured AP for up to 300 weeks. The Michigan and Utah electrodes are currently the most widely used microelectrodes (Figure 4).

The first silicon-based neural electrode—the so-called “Michigan” electrode—was developed in 1970 [46], and fabricated using MEMS processes, such as metal deposition and etching on silicon. The probe length ranged from a few millimeters to centimeters, and could easily be connected to the IC circuit, owing to its similarity of fabrication. Wang et al. succeeded in achieving the longest AP recording of 671 days [68]. During the 1990s, the Utah electrode was developed, on which the needles are vertically positioned on the substrate [51]. It was fabricated through sequential passivation, deposition, and dicing. The electrodes were several hundred microns in height and spaced at intervals of less than 400 µm. Additionally, Utah electrodes were implanted using the pneumatic system, and could withstand long periods of implantation as a result of their biocompatibility and structure [69].

#### 3.2.3. Polymer Electrode

**Figure 5:**
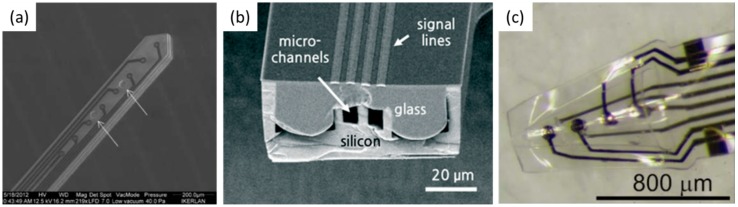
(**a**) Flexible polymer based electrode [76], Copyright 2013, Nature Publication; (**b**) Polymer electrode with micro channel [81], Copyright 2015, Nature Publication; (**c**) The 3D polymer electrode [82], Copyright 2013, Nature Publication.

Electrodes fabricated on a stiff substrate, such as silicon, trigger inflammatory reactions which contains the glial response, and the diffusion of cytokines during chronic recording between the neural electrode and the brain [72]. This inflammatory reaction acts as an insulator at the boundary, resulting in the electrode failure [4]. To avoid this mechanical mismatch, a polymer-based electrode with a low Young’s modulus, and a stiff electrode coated with a soft material, such as hydrogel, emerged as alternatives [73]. Polymers used as substrates, including flexible polyimide [74], Parylene C [75], and SU-8 [76], are generally biocompatible, with a Young’s modulus of only a few GPa (Figure 5). Vitale et al., design a PDMS based CNT electrode having lower impedance per area than carbon fiber [77]. In particular, polymer electrodes have been combined with micro channels. John et al. combined the high-density 3D electrode with the microchannel for local drug delivery [78]. To compensate for the positioning errors that occur because of the flexibility, Wu et al. proposed a fish-bone-shaped electrode [79]. Lu et al., fabricated the flexible electrode based on graphene and confirm no delamination during 30 days [80].

#### 3.2.4. Multifunctional Electrode

In addition to changing the material and its manufacturing method to overcome the limitations of in vivo electrodes such as biocompatibility, there have also been reported cases in which additional functions to solve them. A drug delivery system is installed in the electrode to inject drugs locally. Biotinylated dextran amine (BDA), which is a neuronal tracer used both for anterograde and retrograde, is delivered locally using iontophoresis to find out the recording position [83]. Also, after dexamethasone, an anti-inflammatory drug, was loaded in the conducting polymer to inject only the electrode site, the signal was measured for more than 12 weeks [84]. To measure neurotransmitters like dopamine, which change fast, the fast-scan cyclic voltammetry (FSCV) is suitable. Because the small electrode that has good electrochemical properties is favorable to FSCV, carbon-fiber electrodes are applied in vivo [85]. Electronic devices are also integrated to the electrode. the pre-amplifiers are introduced to boost the neural signals [86], and switching electronics are inserted to adjust recording position without moving the electrode [87].

### 3.3. Requirements and Future Directions

The ultimate goal of the invasive neural electrode is to record the brain’s AP safely, over a long period of time. However, unlike non-invasive electrodes, invasive electrodes must be surgically implanted, and require direct contact with the brain; as such, they necessitate consideration of a greater number of factors than must be considered in relation to non-invasive electrodes. First, the material that comes into contact with the brain should be biocompatible, and this depends critically on the material’s toxicity. Silver, copper are known to be toxic, while platinum, gold are reported to be safe [88]. Also, ceramic material like highly doped poly silicon is used [89]. Recently, conductive polymers have been used as non-toxic materials, with poly 3,4-ethylenedioxythiophene (PEDOT) [90,91], polypyrrole (PPy) [92], and polyaniline (PANI) [93] as typical examples. The mechanical properties of the material are also important. Generally speaking, the Young’s modulus of the brain is less than 10 kPa, but that of the silicon used in microelectrodes is very high, at around 130–185 GPa. The Young’s modulus of polymers is usually only a few GPa—much lower than that of silicon, but still higher than that of the brain. To lower the Young’s modulus significantly, an electrode may be coated with hydrogel, which has a similar Young’s modulus to that of the brain [90]. Also, the shape memory polymers are introduced to solve the mechanical mismatch problem. Simon et al. succeed single unit recording for 77days with the shape memory polymer based electrode whose Young’s modulus is from ~2 GPa to ~50 MPa [94]. In addition, due to the fluidity of the brain, the position of the electrodes can be changed in the macroscale chronically, which means that the spatial resolution of the signal is deteriorated. To improve it, a movable electrode is being developed to solve this problem [95,96].

It is likely that perfectly biocompatible polymer-based neural electrodes will be widely used in the future. During the surgical procedure, preventing the buckling is advantageous to determine the recording position. However, after surgery, the young’s modulus should be reduced by swelling to prevent mechanical mismatch. Hydrogels that retain these characteristics for a long time are preferred. Also, extremely small and flexible electrodes can also be a solution.

## 4. Non-Penetrating Electrodes

Neural electrodes may be classified into penetrating and non-penetrating electrodes. Electrodes currently have the capacity to record neural activity in vivo, from intracellular potential through extracellular APs to LFPs [97]. When these neural signals are applied to BMIs, parity of performance can be achieved between AP and LFP signals with high frequencies (>200 Hz). These signals transmit more information concerning movement parameters, in comparison with EEG signals [98]. The information transmitted by neuronal signals that are extracted from EEG, following the elimination of noise generated from external equipment and movement artifacts, differs in several respects to that obtained from invasive signals [99]. First, there is a disparity in the number and type of neurons: smaller neuronal clusters are recorded, with a lower SNR. Second, the signal composition also varies: since the signals travel a greater distance from the neuron cell to the electrode, the temporal consistency across signal components may be disrupted, and the frequency phase shift may be stronger [100]. Third, there is a difference in the spatial distortion that occurs: neural signals are transferred to the cerebrospinal fluid, skull, and scalp before reaching the EEG electrodes, resulting in a large spatial distortion. These limitations are inherent in the EEG, and cannot be overcome in practice. However, EEG boasts the significant advantage of being able to monitor large-scale connection activities across the entire brain, risk-free and at a lower cost. Invasive electrodes can measure signals that are transmitted deeper in the brain, but they cannot cover the entire cortex and are more difficult to obtain initially, owing to the requisite surgical procedure. On the other hand, electrocorticogram (ECoG), occasionally called intracranial EEG, involves a surgical procedure but does not cause direct brain damage. The neural signals recorded from ECoG electrodes provide much better spatial resolution [101,102] and SNR [103,104] than do EEG electrodes because ECoG electrodes record neural signals directly from the cortical surface.

### 4.1. EEG Electrode

#### 4.1.1. History

In 1875, Richard Caton, a physician practicing in Liverpool, discovered the presence of electrical currents in the brain. His findings concerned the electrical phenomena occurring in the exposed cerebral hemispheres of rabbits and monkeys, and were published in the *British Medical Journal*. In 1890, Adolf Beck reported spontaneous electrical activity and rhythmic oscillations in the brains of rabbits and dogs, altered by light. Beck concluded that fluctuating brain activity is attributable to brain waves [105]. In 1924, Hans Berger, a German physiologist and psychiatrist, first recorded the human brain’s electrical activity on the scalp, using ordinary radio equipment to amplify the electrical signals [106]. His findings are regarded as among the most surprising, remarkable, and momentous developments in the history of clinical neurology. He coined the term ‘electroencephalogram (EEG)’ to describe the electrical potentials in the human brain, and reported that brain activity changed consistently in association with changes in the subject’s general status, e.g., from relaxation to alertness [107]. His findings were later verified by Adrian and Matthews, in 1934, when they identified regular oscillations of 10 to 12 Hz, which they called “alpha rhythms” [108]. In 1947, the American EEG Society was founded, and the first international EEG congress was held. During the 1950s, William Grey Walter developed EEG topography, which facilitated the mapping of electrical activity on the brain’s surface. In 1988, the first robot to be controlled by recorded EEG signals was created and various studies in BMI using EEG signals were reported, including the brain-controlled wheelchair [109] and the robotic arm [110].

#### 4.1.2. EEG Electrodes

EEG electrodes are used to capture changes in the brain’s electrical activity without the need for a surgical procedure. In this review, EEG electrodes were classified into wet, dry and non-contact.

##### Wet EEG Electrodes

Traditional wet EEG electrodes are fabricated from various materials including gold (Au), platinum (Pt), silver/silver-chloride (Ag/AgCl), tin (Sn), and stainless steel (SUS). In 2005, Tallgren et al. concluded that Ag/AgCl electrodes yielded the best performance, with excellent direct current (DC) stability, low noise level, and low resistance among the various materials [111]. Equipment for EEG recording consists of an amplifier unit, an electrode cap, conductive gels, a syringe, and disinfectant. The conductive gels ensure contact between the electrode and the scalp, facilitating the recording of good EEG signals. The wet EEG electrodes may be classified into disposable surface electrodes, reusable disc-electrodes, and saline-based electrodes. Disposable surface electrodes are the simplest and most cost-efficient method of recording EEG signals, and are widely used in other electrophysiological fields, such as electrocardiography (ECG) and electromyography (EMG). However, a major limitation of this method is that the electrode cannot easily be placed on the scalp past the hair, owing to its large size. The reusable disc-electrode overcomes this limitation by reducing the electrode size. The saline-based electrode consists of a sponge and the electrode housing, and the sponge placed on the skin should be soaked with saline during recording.

##### Dry EEG Electrodes

**Figure 6:**
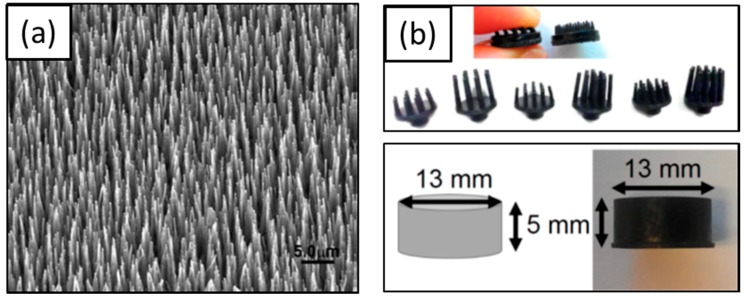
Invasive dry EEG electrodes. (**a**) MWCNT array [115], Copyright 2008, Elsevier; (**b**) pin-shaped (up) and cylinder-shaped conductive polymer dry electrodes (down) [116], Copyright 2014, MDPI.

**Figure 7:**
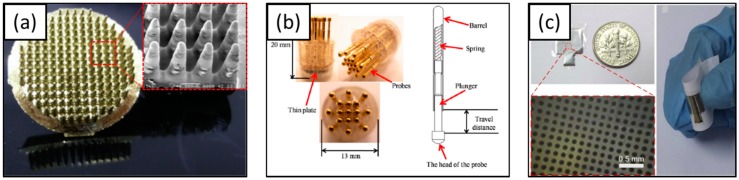
Non-invasive dry EEG electrodes (**a**) 3D printed electrode [117], Copyright 2012, Elsevier; (**b**) dry electrode with 17spring contact probes [120], Copyright 2011, MDPI; (**c**) flexible dry electrode [119], Copyright 2016, Elsevier.

Dry EEG electrodes were introduced to overcome the drawbacks associated with adhesive matter, such as interference between electrodes, dirt on hair and scalp, and the amount of time required to reduce the impedance to acceptable values [112]. These dry EEG electrodes may be sub-categorized into invasive EEG electrodes and non-invasive EEG electrodes. The invasive EEG electrodes have spike array pillars that make direct contact with the scalp, or penetrate slightly into the epidermis of the brain. The microscale pillars may be made of a variety of materials such as silicon [113,114], carbon nanotube (CNT) [115], titanium, or conductive polymer (CP) [116] (Figure 6). These invasive EEG electrodes have been used for chronic experiments in brain-computer interface (BCI) and BMI. However, they can cause skin or tissue reactions triggered by immune response, and the impedance may be mismatched when the pillars are broken. To circumvent these issues, non-invasive EEG electrodes fabricated using micro- or macro-scale conductive pins have been developed. They are convenient to use and can increase the contact area to reduce impedance without penetration. Chen et al. introduced flexible polymer electrodes fabricated with ethylene propylene diene monomer rubber [116], and Salvo et al. fabricated titanium/gold-coated electrodes using a 3D printer [117]. Liao et al. developed dry electrodes with 17 spring contact probes [118] and Peng et al. developed a parylene-based thin and flexible electrode [119] (Figure 7).

### 4.2. ECoG Electrode

ECoG electrodes offer an attractive signal-acquisition method because they do not directly damage the brain. ECoG electrodes are less invasive than penetrating electrodes and have better signal quality, such as spatial resolution and SNR, than EEG electrodes. Thus, ECoG electrodes are widely used in brain–machine interface to improve quality of life [121,122,123]. ECoG electrodes are usually 1–2.3 mm in diameter, with a 10-mm inter-electrode distance, and are embedded in a 0.4–0.6-mm-thick Silastic^®^ base [124,125]. Smaller electrodes of 70–1500 μm diameter have been developed for dedicated research in humans, with a higher electrode density of a 1–4-mm inter-electrode distance [126,127]. Several groups have developed flexible and stretchable ECoG electrodes based on polymeric materials, such as PDMS [128], polyimide [129], and Parylene-C [130]. These developments have evolved with advances in material science and flexible electronics and have overcome the drawbacks of traditional penetrating electrodes made from rigid material. The advances in flexible electronics are discussed in more detail in the next paragraph.

### 4.3. Recent Neural Electrodes with Flexible and Stretchable Characteristics

**Figure 8:**
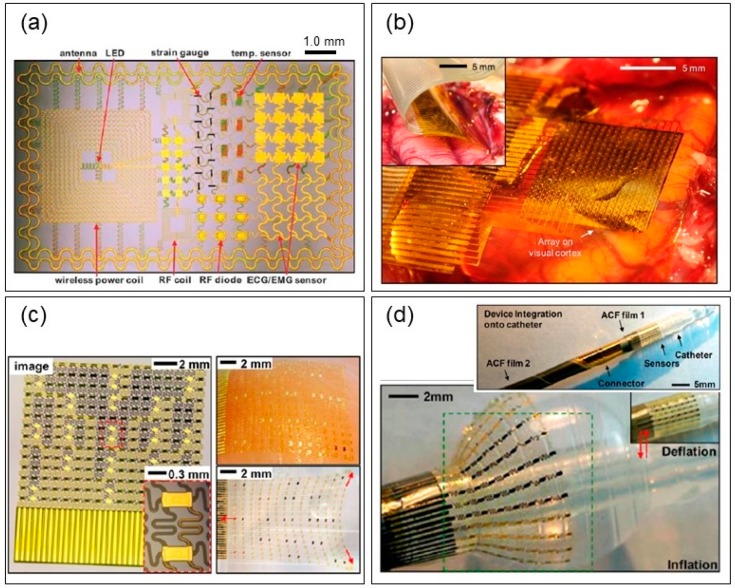
Applications of flexible and stretchable electronics (**a**) Epidermal electronics on PDMS. Image of a demonstration platform for multifunctional electronics and a commercial temporary transfer tattoo onto skin [137], Copyright 2011, Science Publication; (**b**) brain‒machine interface on polyamide. Electrode arrays placed on the visual cortex [141], Copyright 2011, Nature Publication; (**c**) electrocardiogram mapping devices on Ecoflex. Sensor web with no slip page up to ∼22% strain [143], Copyright 2012, National Academy of Sciences; (**d**) smart or minimally invasive surgical tools on PDMS. Multifunctional inflatable balloon catheters [145], Copyright 2011, Nature Publication.

Research into flexible electronics commenced almost 20 years ago [131,132] in response to the demand for macroelectronics [133], such as paper-like flexible displays [134,135]. Flexible and stretchable electronics realized their ultimate application potential in the late 2000s, when the concept of biologically integrated electronics was proposed [136]. They can contribute to the establishment of long-term, intimate bioelectronic interfaces, including epidermal electronics, for vital sign monitoring [137,138,139], BMIs with ECoG [140,141] and EMG [142] electrocardiogram mapping devices [143,144], and smart or minimally invasive surgical tools [145,146] (Figure 8). In applying such bioelectronic interfaces, it is of the utmost importance that they match with the soft and curvilinear tissues of the biological system. In particular, when flexible electronics are applied to BMIs, they must overcome challenges pertaining to the following issues: first, the electrodes should have sufficient performance capacity to detect an electrical potential of several tens µV; second, electrode arrays should have high spatial properties regarding brain activity in vivo; third, the substrate should be ultra-thin to ensure that the electronics impose minimal stresses on the brain; and finally, a high conformal coverage is required between the electrodes and the tissue. Recently, these bioelectronic interfaces have been further combined with wireless transmission, such as Bluetooth and near field communication (NFC) [147,148], and they can also function in an aqueous environment [149]. They have proven useful in diagnosis and homecare systems owing to their good portability and wearability [150]. Bioelectronic interfaces with tripolar concentric rings and capacitive electrodes have recently been introduced, and are placed on the auricle and mastoid, locations that are non-invasive and provide unique electrical isolation, compared with previously used scalp regions, thus improving the SNRs of weak EEG signals [151].

## 5. The Selection of Materials and Surface Modification

Research into neural electrodes has hitherto been conducted across a multitude of aspects, particularly from the material perspective. To measure high-quality vital signals, the electrode must be placed in direct contact with, or implanted into, the cell or anatomical part, and the electrode must be composed of a biocompatible material that does not cause an immune response. Additionally, much research has been conducted with a view to forming various nanostructures on the electrode, and selecting the electrode material for optimum functionality and electrochemical characteristics. The ensuing discussion will examine the types and characteristics of the materials that constitute the electrodes themselves, and surface modification using nanostructures synthesized on the electrode.

### 5.1. The Materials of Substrate and Electrode Parts

Conventional penetrating electrodes were primarily fabricated using rigid materials. Owing to advances in materials engineering, high-density, flat-shaped electrodes, such as EEG and ECoG, have been developed in the form of non-penetrating electrodes. The electrodes used for measuring biological signals should pose no harm to the cells or the human body. As such, it is recommended that the substrate and the metal parts constituting the electrode are composed of materials that do not trigger chronic reactions.

In recent years, several electrodes fabricated on flexible substrates have been developed, and long-term biostability in real recording environments has been studied. The electrode’s composition may be roughly divided into the substrate and the electrode, and this review will adhere to these conventions.

The substrate of the electrode to be implanted in the brain must have sufficient rigidity to facilitate penetration; if it is too rigid, however, it can damage the brain tissue, which has a Young’s modulus of around 3 kPa. It is imperative, therefore, to use a material that has a moderate Young’s modulus. Several soft substrate materials have been found to satisfy these constraints (Table 1), including SU-8 [152,153], polydimethylsiloxane (PDMS) [154], polyimide (PI) [155], and poly(chloro-p-xylylene) (parylene C) [75,156]. The moderate Young’s modulus of these soft materials renders them suitable for use as substrates for fabricating electrodes that can be implanted in the brain without causing damage to the tissue. In this regard, studies are performed to use shape memory polymers (parylene-C + poly(vinyl alchohol) (PVA)) [94], liquid crystal polymers [157], and nanocomposites (cellulose nanofiber network + polyvinyl acetate matrix) [158] that can modulate Young’s modulus as substrate materials. Those studies have aimed at longterm signal recording regardless of the rigidity of the substrate in neural signal recording. In general, neural electrodes that measure brain signals in an in vivo environment were capable of stable signal measurement for several days to several weeks [1,159,160,161,162]. Recently, electrodes made with SU-8 as a substrate material have been used for recording single-unit spikes for up to 8 months [163].

Moreover, the metal used in the electrode should have good electrical conductivity properties for electrical signal transmission, with no toxicity, considering its long-term implantation in the brain. Studies (Table 2) have also been conducted on copper (Cu) [164], gold (Au) [165,166,167], platinum (Pt) [168,169,170,171,172,173], silver (Ag) [58], titanium (Ti) [174], tungsten (W) [175], indium-tin-oxide (ITO) [176] and graphene [177] which are the most commonly used materials used in fabricating electrodes. All these materials have good electrical conductivity properties at room temperature, but copper and silver are toxic when implanted in the brain, rendering them unsuitable for use as in nerve electrodes.

### 5.2. Surface Modification for Enhancing Electrode Impedance

As mentioned above, neural electrodes have been studied extensively, particularly in terms of material. Surface modification using nanostructures is applied to the surfaces of electrodes to enhance their functionality. This section reviews the studies that have been conducted into nanostructure fabrication for improving electrical properties. An important characteristic for consideration in measuring neural signals is low electrical impedance. Generally, neural electrodes have a smaller electrode size, which can increase the spatial resolution of the neural signal (signal selectivity). However, a smaller electrode size is associated with larger electrode impedance, which in turn increases the noise of the signal (signal sensitivity). As such, maximization of the electrochemical surface area (ESA) of electrochemical reactions, while simultaneously maintaining the geometrical surface area (GSA), offers a means of reducing electrical impedance.

**Figure 9:**
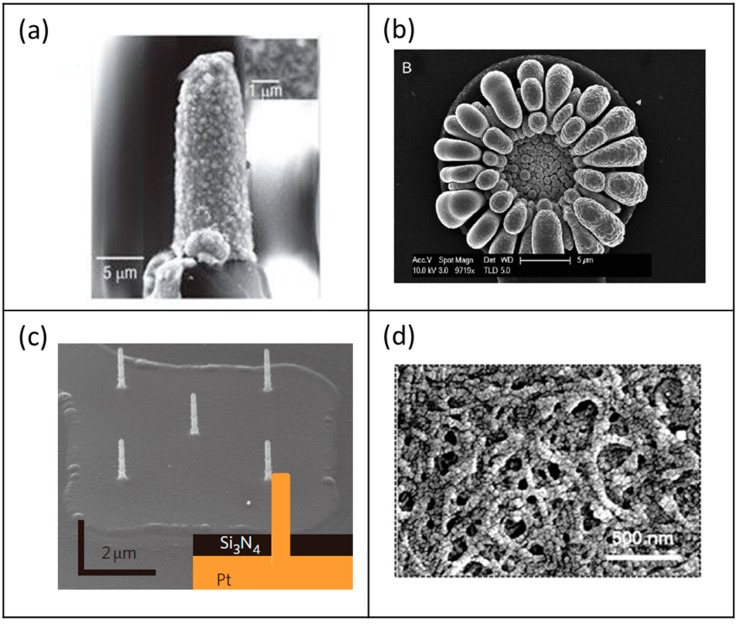
Various surface modification techniques for improved neural signal recording performance. (**a**) Electrode modified with CNTs by electrodeposition [180], Copyright 2008, Nature Publishing; (**b**) electrode modified with gold nanograins [165], Copyright 2013, WILEY-VCH Verlag; (**c**) electrode modified with platinum nanopillars [169], Copyright 2012, Nature Publishing; (**d**) electrode modified with CNT‒Au nanocomposite hierarchical structures [184], Copyright 2017, Elsevier.

The modification of electrode surfaces uses inorganic, organic, and hybrid composite materials (Table 3 and Figure 9). First, inorganic materials may be classified into metal-based coatings, such as platinum black [170] and iridium oxide [178], and metal nanostructure coatings that cover various types of metal nanostructure. Metal nanostructures may be classified into nanopillar [169], nanorod [179], nanoflake [166], and nanograin [165] shapes. They are formed on the surface of the electrode, to maximize the ESA and reduce impedance when the GSA is equivalent.

Second, with regard to organic materials, carbon-based materials, such as CNTs and graphene, have also been investigated as viable options for coating neural electrodes. CNTs may be divided into single-walled nanotubes (SWNTs) [156] and multi-walled nanotubes (MWNTs) [180,181,182,183], and these materials boast multiple advantages, including mechanical stability, ESA versus GSA, electrical properties, chemical stability, and biocompatibility. Graphene also has high electrical conductivity, high elastic modulus and high electron mobility. It can be used as a single layer over a large area, and may be transferred to a soft substrate.

Third, in recent years, studies have sought to moderate electrode impedance by coating the electrodes with hybrid composite materials, in a bid to overcome the limitations associated with single-material coating. Kim et al. [184] also synthesized gold nanoparticles to fabricate the hierarchical structure after coating with CNT. This method may facilitate the fabrication of a neural electrode that maximizes increased ESA without simultaneously increasing GSA.

## 6. Conclusions and Perspectives

Since the advent of neuronal electrodes, many studies have attempted to interpret the signals accepted by a neural electrode. The development of MEMS and micromachining technologies has reduced the size of electrodes, and a variety of designs and materials have been created to record many selective, low-noise signals. Here, we divided the neuronal signal into three types: the MEA electrode, which records neuronal signals in vitro; the penetrating-type electrode, which measures signals in vivo, particularly in the brain; and the non-penetrating type, which records from on or beneath the scalp. We examined the main issues related to the neural electrode: sensitivity (SNR), selectivity, and biocompatibility. The density of all electrode types has been increased, and various functions have been added. Base materials are becoming soft and stretchable to match the mechanical properties of human tissue, and electrode interfaces require higher conductivity and biocompatibility. Additionally, various electronic devices have been added, and functional strategies, such those for drug delivery, have been introduced. The electrode interfaces have been applied to new materials, such as conductive polymers and nanomaterials. Through these advances, neural electrodes have improved sensitivity and selectivity and have increased the recording period by improving biocompatibility.

The greatest demand for neural interface technology is the BMI, which connects the brain and a machine. This technology provides new hope to those who have lost part of their body by enabling the user to manipulate the artificial body through a neural interface. This technology is emerging as a new paradigm for input devices, such as keyboards and touchpads. However, some challenges remain, such as an inadequate understanding of the brain and its mechanisms, the chronic separation of adhesive electrodes, a low SNR, and inflammation associated with invasive electrodes. The previous decades of research and effort will allow us to realize the perfect neural interface.

## Figures and Tables

**Table 1:** Properties of substrate materials.

Electrode Material	Young’s Modulus (GPa)	Toxicity	Reference
SU-8	2.0	Non-toxic	[152,153]
PDMS	0.00132–0.00297	Non-toxic	[154]
PI	2.5	Non-toxic	[155]
Parylene C	2.76	Non-toxic	[75,156]

**Table 2:** Properties of electrode materials.

Electrode Material	Electrical Conductivity (at 20 °C, S/m)	Toxicity	Reference
Copper (Cu)	5.96 × 10^7^	Toxic	[164]
Gold (Au)	4.10 × 10^7^	Non-toxic	[165,166,167]
Platinum (Pt)	9.43 × 10^6^	Non-toxic	[168,169,170,171,172,173]
Silver (Ag)	6.30 × 10^7^	Toxic	[58]
Titanium (Ti)	2.38 × 10^6^	Non-toxic	[174]
Tungsten (W)	1.79 × 10^7^	Non-toxic	[175]
Indium-tin-oxide	1.3 × 10^4^	Non-toxic	[176]
Graphene	1.0 × 10^2^	Non-toxic	[177]

**Table 3:** Coating materials and surface modification techniques for enhancing electrode impedance.

Material	Surface Modification Technique	References
Inorganic	Metal coating	[170,178]
	Metal nanostructure coating	[165,167,169,179]
Organic	Carbon based material coating	[156,180,181,182,183]
Hybrid	Composite material coating	[184]
